# The Dissection Bill

**Published:** 1854-04

**Authors:** 


*The Dissection Bill.*—This bill is still dragging its slow length through the Assembly. We are told, by those who are capable of forming an opinion, that it will be passed. There has been some able debate upon it, and the arguments in its favor have been very manfully supported by its friends. When it gets through its embryonic existence and becomes a law of the land, we shall have some fault to find with it, but for the present we are only anxious for its passage.

